# Crystal structure of di­aqua­bis­(2,6-di­methyl­pyrazine-κ*N*)bis­(thio­cyanato-κ*N*)cobalt(II) 2,5-di­methyl­pyrazine tris­olvate

**DOI:** 10.1107/S2056989015024184

**Published:** 2015-12-24

**Authors:** Stefan Suckert, Susanne Wöhlert, Inke Jess, Christian Näther

**Affiliations:** a Institut für Anorganische Chemie, Christian-Albrechts-Universität Kiel, Max-Eyth-Strasse 2, 24118 Kiel, Germany

**Keywords:** crystal structure, coordination compound, octa­hedral coordination, cobalt(II), di­methyl­pyrazine

## Abstract

In the crystal structure of the title compound, [Co(NCS)_2_(C_6_H_8_N_2_)_2_(H_2_O)_2_]·3C_6_H_8_N_2_, the Co^II^ cation is coordinated by two terminally *N*-bound thio­cyanate anions, two water mol­ecules and two 2,6-di­methyl­pyrazine ligands, forming a discrete complex with a slightly distorted octa­hedral N_4_O_2_ coordination environment. The asymmetric unit contains one Co^II^ cation and three halves of 2,5-di­methyl­pyrazine solvate mol­ecules, all entities being completed by inversion symmetry, as well as one thio­cyanate anion, an aqua ligand and a 2,6-di­methyl­pyrazine ligand, all in general positions. In the crystal, discrete complexes are arranged in a way that cavities are formed where the noncoordinating 2,5-di­methyl­pyrazine mol­ecules are located. The coordination of the latter to the metal is prevented due to the bulky methyl groups in vicinal positions to the N atoms, leading to a preferential coordination through the 2,6-di­methyl­pyrazine ligands. The complex mol­ecules are linked by O—H⋯N hydrogen bonds between the water H atoms and the N atoms of 2,5-di­methyl­pyrazine solvent mol­ecules, leading to a layered structure extending parallel to (100).

## Related literature   

The crystal structure of the 2,5-di­methyl­pyrazine monosolvate of the title compound was reported recently (Suckert *et al.*, 2015*b* ▸). For the structures of other metal thio­cyanates with 2,5-di­methyl­pyrazine or 2,6-di­methyl­pyrazine, see: Otieno *et al.* (2003 ▸); Mahmoudi & Morsali (2009 ▸); Suckert *et al.* (2015*a* ▸).
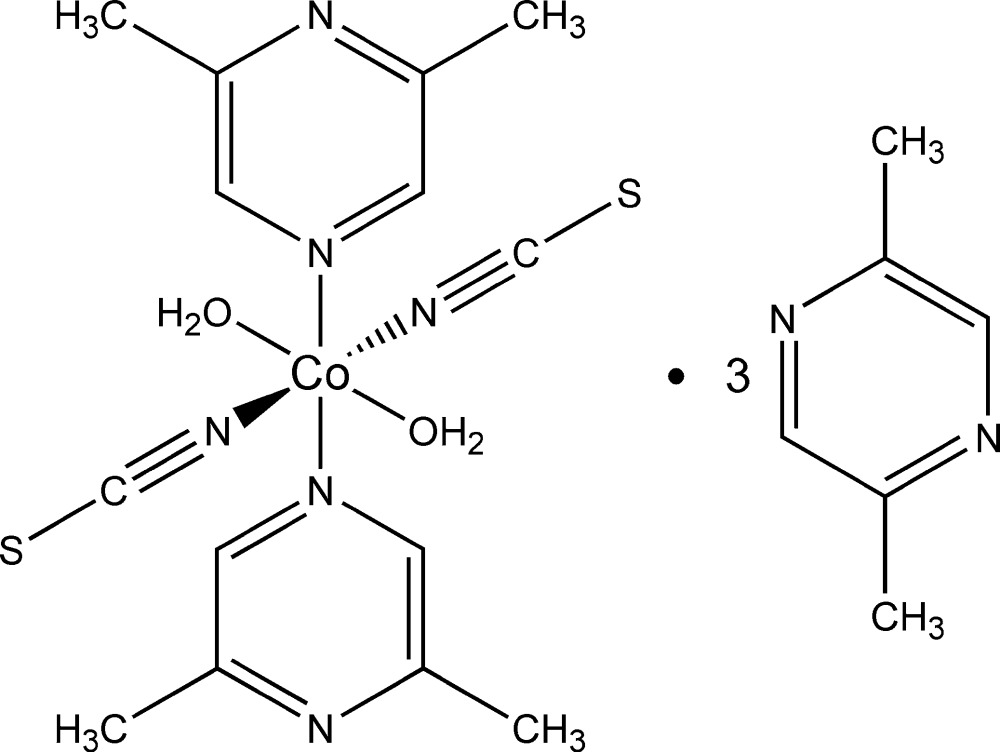


## Experimental   

### Crystal data  

[Co(NCS)_2_(C_6_H_8_N_2_)_2_(H_2_O)_2_]·C_6_H_8_N_2_
*M* *_r_* = 751.84
Triclinic, 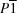

*a* = 9.3296 (8) Å
*b* = 10.8407 (8) Å
*c* = 11.3906 (9) Å
α = 103.231 (9)°
β = 111.888 (9)°
γ = 104.123 (9)°
*V* = 968.99 (15) Å^3^
*Z* = 1
Mo *K*α radiation
μ = 0.60 mm^−1^
*T* = 200 K
0.17 × 0.13 × 0.06 mm

### Data collection  

Stoe IPDS-1 diffractometer
Absorption correction: numerical (*X-SHAPE* and *X-RED32*; Stoe, 2008 ▸) *T*_min_ = 0.909, *T*_max_ = 0.963
8838 measured reflections
4092 independent reflections
3540 reflections with *I* > 2σ(*I*)
*R*_int_ = 0.049

### Refinement  

*R*[*F* ^2^ > 2σ(*F* ^2^)] = 0.047
*wR*(*F* ^2^) = 0.130
*S* = 1.05
4092 reflections
228 parameters
H-atom parameters constrained
Δρ_max_ = 0.37 e Å^−3^
Δρ_min_ = −0.79 e Å^−3^

### 

Data collection: *X-AREA* (Stoe, 2008 ▸); cell refinement: *X-AREA*; data reduction: *X-AREA*; program(s) used to solve structure: *SHELXS97* (Sheldrick, 2008 ▸); program(s) used to refine structure: *SHELXL2013* (Sheldrick, 2015 ▸); molecular graphics: *XP* in *SHELXTL* (Sheldrick, 2008 ▸) and *DIAMOND* (Brandenburg, 1999 ▸); software used to prepare material for publication: *publCIF* (Westrip, 2010 ▸).

## Supplementary Material

Crystal structure: contains datablock(s) I, global. DOI: 10.1107/S2056989015024184/wm5255sup1.cif

**Fig. 1.**
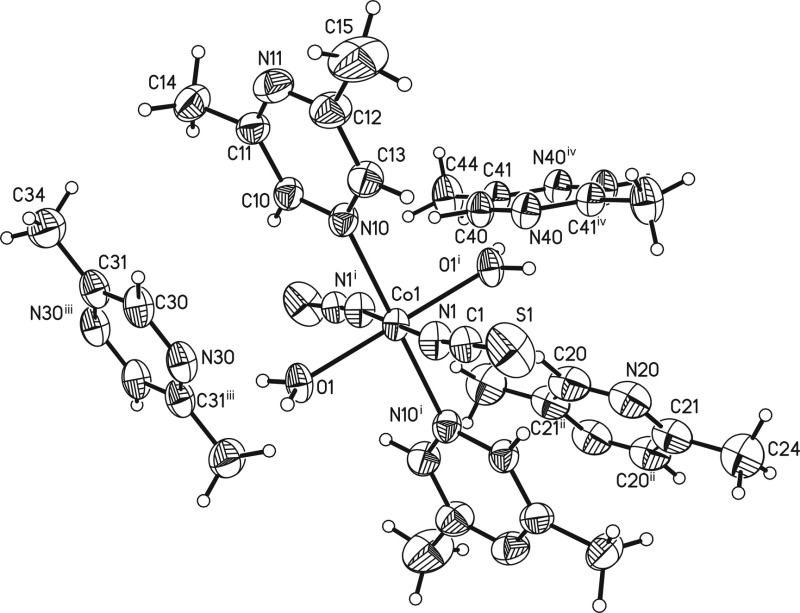
The structures of the molecular entities of the title compound. Displacement ellipsoids are drawn at the 50% probability level. [Symmetry codes: (i) -x + 1, y + 1, -z + 1; (ii) -x + 1, -y + 1, -z; (iii) -x + 1, -y + 2, -z + 1; (iv) -x + 1, -y + 1, -z + 2.]

Structure factors: contains datablock(s) I. DOI: 10.1107/S2056989015024184/wm5255Isup2.hkl

x y z x y z x y z x y z . DOI: 10.1107/S2056989015024184/wm5255fig1.tif
The structures of the mol­ecular entities of the title compound. Displacement ellipsoids are drawn at the 50% probability level. [Symmetry codes: (i) −*x* + 1, *y* + 1, −*z* + 1; (ii) −*x* + 1, −*y* + 1, −*z*; (iii) −*x* + 1, −*y* + 2, −*z* + 1; (iv) −*x* + 1, −*y* + 1, −*z* + 2.]

**Fig. 2.**
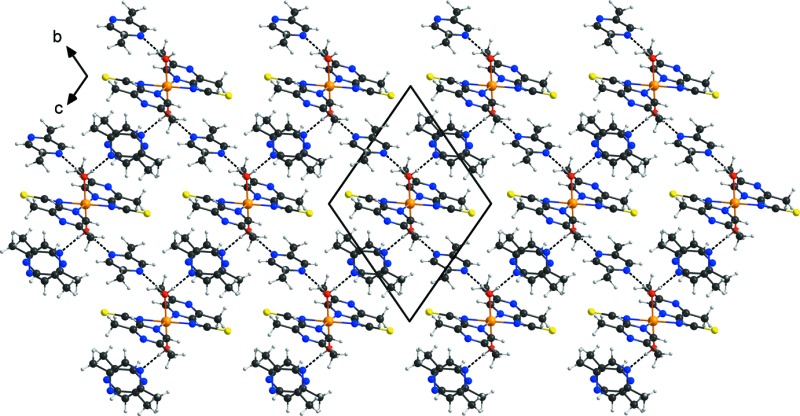
The crystal packing of the title compound in a view along [100]. Hydrogen bonding is shown as dashed lines.

. DOI: 10.1107/S2056989015024184/wm5255fig2.tif
The crystal packing of the title compound in a view along [100]. Hydrogen bonding is shown as dashed lines.

CCDC reference: 1442780

Additional supporting information:  crystallographic information; 3D view; checkCIF report

## Figures and Tables

**Table 1:** Hydrogen-bond geometry (Å, °)

*D*—H⋯*A*	*D*—H	H⋯*A*	*D*⋯*A*	*D*—H⋯*A*
O1—H1*O*1⋯N30	0.82	1.98	2.796 (2)	172
O1—H2*O*1⋯N40^i^	0.82	2.00	2.816 (2)	174

Symmetry code: (i) 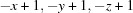.

## supplementary crystallographic information

### S1. Synthesis and crystallization

Co(SCN)_2_ and 2,5-di­methyl­pyrazine (97%) were purchased from Alfa Aesar. The title compound was prepared by the reaction of 28.9 mg (0.15 mmol) Co(NCS)_2_·H_2_O in 195.0 µl (1.8 mmol) 2,5-di­methyl­pyrazine at room temperature. After a few days, plate-like crystals of the title compound were obtained that contained 2,6-di­methyl­pyrazine in addition. Later it was found that the commercially available 2,5-di­methyl­pyrazine contains about 3%_wt_ of 2,6-di­methyl­pyrazine as a contamination.

### S2. Refinement

C-bound hydrogen atoms were positioned with idealized geometry (methyl H atoms were allowed to rotate but not to tip) and were refined with *U*_eq_(H) = 1.2*U*_eq_(C) (1.5 for methyl H atoms) using a riding model with C—H = 0.95 Å for aromatic H atoms and C—H = 0.98 Å for methyl H atoms. The O-bound hydrogen atoms were located in a difference map. The O—H bond length was constrained to 0.84 Å and H atoms were refined with *U*_iso_(H) = 1.5*U*_eq_(O) using a riding model.

### Crystal data

**Table:**

[Co(NCS)_2_(C_6_H_8_N_2_)_2_(H_2_O)_2_]·C_6_H_8_N_2_	*Z* = 1
*M**_r_* = 751.84	*F*(000) = 395
Triclinic, *P*1	*D*_x_ = 1.288 Mg m^−^^3^
*a* = 9.3296 (8) Å	Mo *K*α radiation, λ = 0.71073 Å
*b* = 10.8407 (8) Å	Cell parameters from 8844 reflections
*c* = 11.3906 (9) Å	θ = 2.6–22.0°
α = 103.231 (9)°	µ = 0.60 mm^−^^1^
β = 111.888 (9)°	*T* = 200 K
γ = 104.123 (9)°	Plate, purple
*V* = 968.99 (15) Å^3^	0.17 × 0.13 × 0.06 mm

### Data collection

**Table:**

Stoe IPDS-1 diffractometer	3540 reflections with *I* > 2σ(*I*)
Radiation source: fine-focus sealed tube	*R*_int_ = 0.049
phi scans	θ_max_ = 27.4°, θ_min_ = 2.6°
Absorption correction: numerical (*X-SHAPE* and *X-RED32*; Stoe, 2008)	*h* = −11→11
*T*_min_ = 0.909, *T*_max_ = 0.963	*k* = −13→13
8838 measured reflections	*l* = −14→14
4092 independent reflections	

### Refinement

**Table:**

Refinement on *F*^2^	0 restraints
Least-squares matrix: full	Hydrogen site location: mixed
*R*[*F*^2^ > 2σ(*F*^2^)] = 0.047	H-atom parameters constrained
*wR*(*F*^2^) = 0.130	*w* = 1/[σ^2^(*F*_o_^2^) + (0.0772*P*)^2^ + 0.2726*P*] where *P* = (*F*_o_^2^ + 2*F*_c_^2^)/3
*S* = 1.05	(Δ/σ)_max_ = 0.032
4092 reflections	Δρ_max_ = 0.37 e Å^−^^3^
228 parameters	Δρ_min_ = −0.79 e Å^−^^3^

### Special details

**Table:**

Geometry. All esds (except the esd in the dihedral angle between two l.s. planes) are estimated using the full covariance matrix. The cell esds are taken into account individually in the estimation of esds in distances, angles and torsion angles; correlations between esds in cell parameters are only used when they are defined by crystal symmetry. An approximate (isotropic) treatment of cell esds is used for estimating esds involving l.s. planes.

### Fractional atomic coordinates and isotropic or equivalent isotropic displacement parameters (Å^2^)

**Table:**

	*x*	*y*	*z*	*U*_iso_*/*U*_eq_	
Co1	0.5000	0.5000	0.5000	0.02727 (14)	
N1	0.4584 (2)	0.32856 (19)	0.34740 (18)	0.0352 (4)	
C1	0.4289 (3)	0.2251 (2)	0.2698 (2)	0.0337 (4)	
S1	0.38697 (12)	0.08006 (6)	0.15982 (8)	0.0628 (2)	
N10	0.2366 (2)	0.47166 (18)	0.40932 (18)	0.0339 (4)	
C10	0.1832 (3)	0.5739 (2)	0.4318 (2)	0.0351 (4)	
H10	0.2616	0.6618	0.4927	0.042*	
C11	0.0166 (3)	0.5558 (3)	0.3691 (2)	0.0397 (5)	
C12	−0.0429 (3)	0.3307 (3)	0.2646 (3)	0.0492 (6)	
C13	0.1238 (3)	0.3506 (2)	0.3258 (2)	0.0416 (5)	
H13	0.1579	0.2757	0.3073	0.050*	
C14	−0.0406 (3)	0.6725 (3)	0.3916 (3)	0.0533 (6)	
H14A	−0.0693	0.6993	0.3118	0.080*	
H14B	0.0483	0.7496	0.4710	0.080*	
H14C	−0.1384	0.6451	0.4069	0.080*	
C15	−0.1686 (4)	0.1922 (4)	0.1715 (4)	0.0830 (12)	
H15A	−0.2500	0.1654	0.2047	0.125*	
H15B	−0.1129	0.1265	0.1684	0.125*	
H15C	−0.2252	0.1939	0.0804	0.125*	
N11	−0.0949 (2)	0.4341 (2)	0.2867 (2)	0.0482 (5)	
C20	0.8651 (3)	0.4951 (3)	0.9060 (3)	0.0539 (7)	
H20	0.7663	0.4936	0.8381	0.065*	
N20	0.8981 (3)	0.3820 (3)	0.8896 (2)	0.0532 (6)	
C24	1.0768 (4)	0.2597 (4)	0.9700 (4)	0.0691 (8)	
H24A	0.9786	0.1815	0.9460	0.104*	
H24B	1.1671	0.2702	1.0554	0.104*	
H24C	1.1115	0.2448	0.8982	0.104*	
N30	0.5186 (3)	0.87945 (19)	0.4487 (2)	0.0425 (4)	
C30	0.3732 (3)	0.8941 (2)	0.4031 (2)	0.0431 (5)	
H30	0.2788	0.8192	0.3329	0.052*	
C31	0.3532 (3)	1.0149 (2)	0.4541 (2)	0.0423 (5)	
C21	1.0360 (3)	0.3855 (3)	0.9857 (3)	0.0515 (6)	
C34	0.1905 (4)	1.0312 (3)	0.4020 (4)	0.0596 (7)	
H34A	0.1321	1.0032	0.4529	0.089*	
H34B	0.1241	0.9743	0.3059	0.089*	
H34C	0.2076	1.1267	0.4126	0.089*	
N40	0.4624 (3)	0.4506 (2)	0.86521 (18)	0.0390 (4)	
C40	0.4770 (3)	0.5764 (2)	0.9248 (2)	0.0398 (5)	
H40	0.4610	0.6333	0.8726	0.048*	
C41	0.5148 (3)	0.6284 (2)	1.0603 (2)	0.0369 (4)	
C44	0.5314 (4)	0.7707 (3)	1.1266 (3)	0.0566 (7)	
H44A	0.4322	0.7690	1.1384	0.085*	
H44B	0.5434	0.8240	1.0697	0.085*	
H44C	0.6294	0.8122	1.2152	0.085*	
O1	0.5467 (2)	0.62397 (14)	0.39186 (14)	0.0351 (3)	
H1O1	0.5451	0.7009	0.4059	0.053*	
H2O1	0.5481	0.5992	0.3192	0.053*	

### Atomic displacement parameters (Å^2^)

**Table:**

	*U*^11^	*U*^22^	*U*^33^	*U*^12^	*U*^13^	*U*^23^
Co1	0.0321 (2)	0.02219 (19)	0.0215 (2)	0.00973 (14)	0.00805 (14)	0.00454 (14)
N1	0.0432 (9)	0.0284 (8)	0.0262 (8)	0.0120 (7)	0.0122 (7)	0.0035 (7)
C1	0.0383 (10)	0.0302 (10)	0.0301 (10)	0.0121 (8)	0.0140 (8)	0.0094 (8)
S1	0.0962 (6)	0.0294 (3)	0.0568 (4)	0.0147 (3)	0.0435 (4)	−0.0014 (3)
N10	0.0333 (8)	0.0325 (9)	0.0306 (8)	0.0121 (7)	0.0091 (7)	0.0114 (7)
C10	0.0354 (10)	0.0360 (10)	0.0334 (10)	0.0142 (8)	0.0129 (8)	0.0145 (9)
C11	0.0358 (10)	0.0493 (13)	0.0389 (11)	0.0175 (9)	0.0186 (9)	0.0196 (10)
C12	0.0354 (11)	0.0461 (13)	0.0484 (14)	0.0073 (10)	0.0107 (10)	0.0086 (11)
C13	0.0364 (11)	0.0353 (11)	0.0411 (12)	0.0093 (9)	0.0104 (9)	0.0094 (9)
C14	0.0482 (13)	0.0617 (16)	0.0614 (16)	0.0317 (12)	0.0263 (12)	0.0268 (14)
C15	0.0432 (15)	0.0558 (18)	0.094 (3)	−0.0011 (13)	0.0064 (16)	−0.0079 (18)
N11	0.0344 (9)	0.0547 (12)	0.0478 (11)	0.0135 (9)	0.0140 (8)	0.0158 (10)
C20	0.0376 (12)	0.0704 (18)	0.0329 (12)	0.0066 (12)	0.0059 (9)	0.0141 (12)
N20	0.0433 (11)	0.0593 (14)	0.0330 (10)	0.0024 (10)	0.0077 (8)	0.0089 (10)
C24	0.0691 (19)	0.072 (2)	0.0572 (18)	0.0237 (16)	0.0231 (15)	0.0190 (16)
N30	0.0653 (12)	0.0271 (8)	0.0355 (10)	0.0184 (9)	0.0236 (9)	0.0090 (8)
C30	0.0606 (14)	0.0251 (9)	0.0374 (11)	0.0145 (9)	0.0182 (10)	0.0084 (9)
C31	0.0601 (14)	0.0296 (10)	0.0404 (12)	0.0175 (10)	0.0249 (11)	0.0132 (9)
C21	0.0431 (12)	0.0624 (16)	0.0350 (12)	0.0073 (11)	0.0122 (10)	0.0154 (11)
C34	0.0575 (15)	0.0407 (13)	0.078 (2)	0.0197 (12)	0.0283 (15)	0.0200 (14)
N40	0.0508 (10)	0.0414 (10)	0.0277 (8)	0.0217 (8)	0.0174 (8)	0.0128 (8)
C40	0.0538 (12)	0.0402 (11)	0.0320 (11)	0.0226 (10)	0.0205 (9)	0.0164 (9)
C41	0.0453 (11)	0.0369 (11)	0.0309 (10)	0.0178 (9)	0.0178 (9)	0.0123 (9)
C44	0.090 (2)	0.0413 (13)	0.0464 (14)	0.0312 (14)	0.0351 (14)	0.0140 (12)
O1	0.0549 (9)	0.0257 (7)	0.0277 (7)	0.0181 (6)	0.0194 (6)	0.0101 (6)

### Geometric parameters (Å, º)

**Table:**

Co1—N1^i^	2.0718 (18)	N20—C21	1.327 (4)
Co1—N1	2.0718 (18)	C24—C21	1.494 (5)
Co1—O1	2.0942 (15)	C24—H24A	0.9800
Co1—O1^i^	2.0942 (15)	C24—H24B	0.9800
Co1—N10^i^	2.1895 (18)	C24—H24C	0.9800
Co1—N10	2.1895 (18)	N30—C31^iii^	1.316 (3)
N1—C1	1.153 (3)	N30—C30	1.324 (4)
C1—S1	1.621 (2)	C30—C31	1.393 (3)
N10—C13	1.320 (3)	C30—H30	0.9500
N10—C10	1.333 (3)	C31—N30^iii^	1.316 (3)
C10—C11	1.387 (3)	C31—C34	1.481 (4)
C10—H10	0.9500	C21—C20^ii^	1.373 (4)
C11—N11	1.320 (3)	C34—H34A	0.9800
C11—C14	1.494 (4)	C34—H34B	0.9800
C12—N11	1.335 (4)	C34—H34C	0.9800
C12—C13	1.382 (3)	N40—C40	1.326 (3)
C12—C15	1.497 (4)	N40—C41^iv^	1.336 (3)
C13—H13	0.9500	C40—C41	1.386 (3)
C14—H14A	0.9800	C40—H40	0.9500
C14—H14B	0.9800	C41—N40^iv^	1.336 (3)
C14—H14C	0.9800	C41—C44	1.495 (3)
C15—H15A	0.9800	C44—H44A	0.9800
C15—H15B	0.9800	C44—H44B	0.9800
C15—H15C	0.9800	C44—H44C	0.9800
C20—N20	1.325 (4)	O1—H1O1	0.8175
C20—C21^ii^	1.373 (4)	O1—H2O1	0.8152
C20—H20	0.9500		
			
N1^i^—Co1—N1	180.0	C11—N11—C12	118.4 (2)
N1^i^—Co1—O1	88.87 (7)	N20—C20—C21^ii^	124.4 (3)
N1—Co1—O1	91.13 (7)	N20—C20—H20	117.8
N1^i^—Co1—O1^i^	91.13 (7)	C21^ii^—C20—H20	117.8
N1—Co1—O1^i^	88.87 (7)	C20—N20—C21	117.1 (2)
O1—Co1—O1^i^	180.0	C21—C24—H24A	109.5
N1^i^—Co1—N10^i^	91.60 (7)	C21—C24—H24B	109.5
N1—Co1—N10^i^	88.40 (7)	H24A—C24—H24B	109.5
O1—Co1—N10^i^	88.71 (7)	C21—C24—H24C	109.5
O1^i^—Co1—N10^i^	91.29 (7)	H24A—C24—H24C	109.5
N1^i^—Co1—N10	88.40 (7)	H24B—C24—H24C	109.5
N1—Co1—N10	91.60 (7)	C31^iii^—N30—C30	117.2 (2)
O1—Co1—N10	91.29 (7)	N30—C30—C31	122.6 (2)
O1^i^—Co1—N10	88.71 (7)	N30—C30—H30	118.7
N10^i^—Co1—N10	180.0	C31—C30—H30	118.7
C1—N1—Co1	172.52 (19)	N30^iii^—C31—C30	120.3 (2)
N1—C1—S1	179.6 (2)	N30^iii^—C31—C34	117.4 (2)
C13—N10—C10	117.27 (19)	C30—C31—C34	122.4 (2)
C13—N10—Co1	120.17 (16)	N20—C21—C20^ii^	118.5 (3)
C10—N10—Co1	122.53 (14)	N20—C21—C24	118.3 (3)
N10—C10—C11	122.0 (2)	C20^ii^—C21—C24	123.2 (3)
N10—C10—H10	119.0	C31—C34—H34A	109.5
C11—C10—H10	119.0	C31—C34—H34B	109.5
N11—C11—C10	120.1 (2)	H34A—C34—H34B	109.5
N11—C11—C14	118.6 (2)	C31—C34—H34C	109.5
C10—C11—C14	121.3 (2)	H34A—C34—H34C	109.5
N11—C12—C13	121.0 (2)	H34B—C34—H34C	109.5
N11—C12—C15	118.8 (2)	C40—N40—C41^iv^	118.11 (19)
C13—C12—C15	120.3 (3)	N40—C40—C41	122.6 (2)
N10—C13—C12	121.3 (2)	N40—C40—H40	118.7
N10—C13—H13	119.3	C41—C40—H40	118.7
C12—C13—H13	119.3	N40^iv^—C41—C40	119.3 (2)
C11—C14—H14A	109.5	N40^iv^—C41—C44	118.5 (2)
C11—C14—H14B	109.5	C40—C41—C44	122.2 (2)
H14A—C14—H14B	109.5	C41—C44—H44A	109.5
C11—C14—H14C	109.5	C41—C44—H44B	109.5
H14A—C14—H14C	109.5	H44A—C44—H44B	109.5
H14B—C14—H14C	109.5	C41—C44—H44C	109.5
C12—C15—H15A	109.5	H44A—C44—H44C	109.5
C12—C15—H15B	109.5	H44B—C44—H44C	109.5
H15A—C15—H15B	109.5	Co1—O1—H1O1	125.2
C12—C15—H15C	109.5	Co1—O1—H2O1	126.2
H15A—C15—H15C	109.5	H1O1—O1—H2O1	107.2
H15B—C15—H15C	109.5		

Symmetry codes: (i) −*x*+1, −*y*+1, −*z*+1; (ii) −*x*+2, −*y*+1, −*z*+2; (iii) −*x*+1, −*y*+2, −*z*+1; (iv) −*x*+1, −*y*+1, −*z*+2.

### Hydrogen-bond geometry (Å, º)

**Table:**

*D*—H···*A*	*D*—H	H···*A*	*D*···*A*	*D*—H···*A*
O1—H1*O*1···N30	0.82	1.98	2.796 (2)	172
O1—H2*O*1···N40^i^	0.82	2.00	2.816 (2)	174

Symmetry code: (i) −*x*+1, −*y*+1, −*z*+1.
