# Supplementary figures and images for: Crystal structure of di­aqua­bis­(2,6-di­methyl­pyrazine-κN)bis­(thio­cyanato-κN)cobalt(II) 2,5-di­methyl­pyrazine tris­olvate
Source: Acta Crystallogr E Crystallogr Commun. 2015 Dec 24;71(Pt 12):m269–70. doi: 10.1107/S2056989015024184 (PMC4719868; doi:10.1107/S2056989015024184)

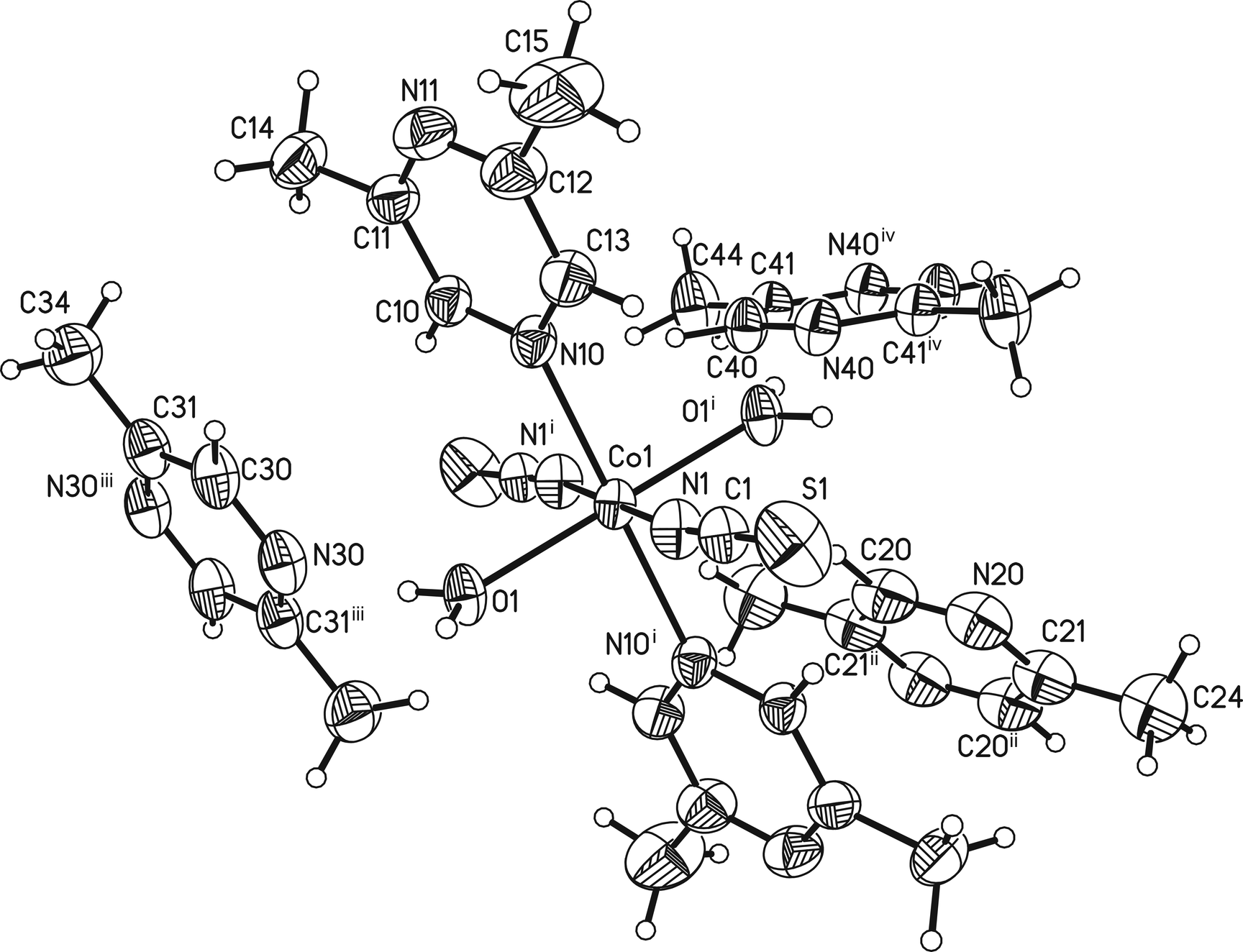

Supplement: Supplementary file 3 [file e-71-0m269-fig1.tif]

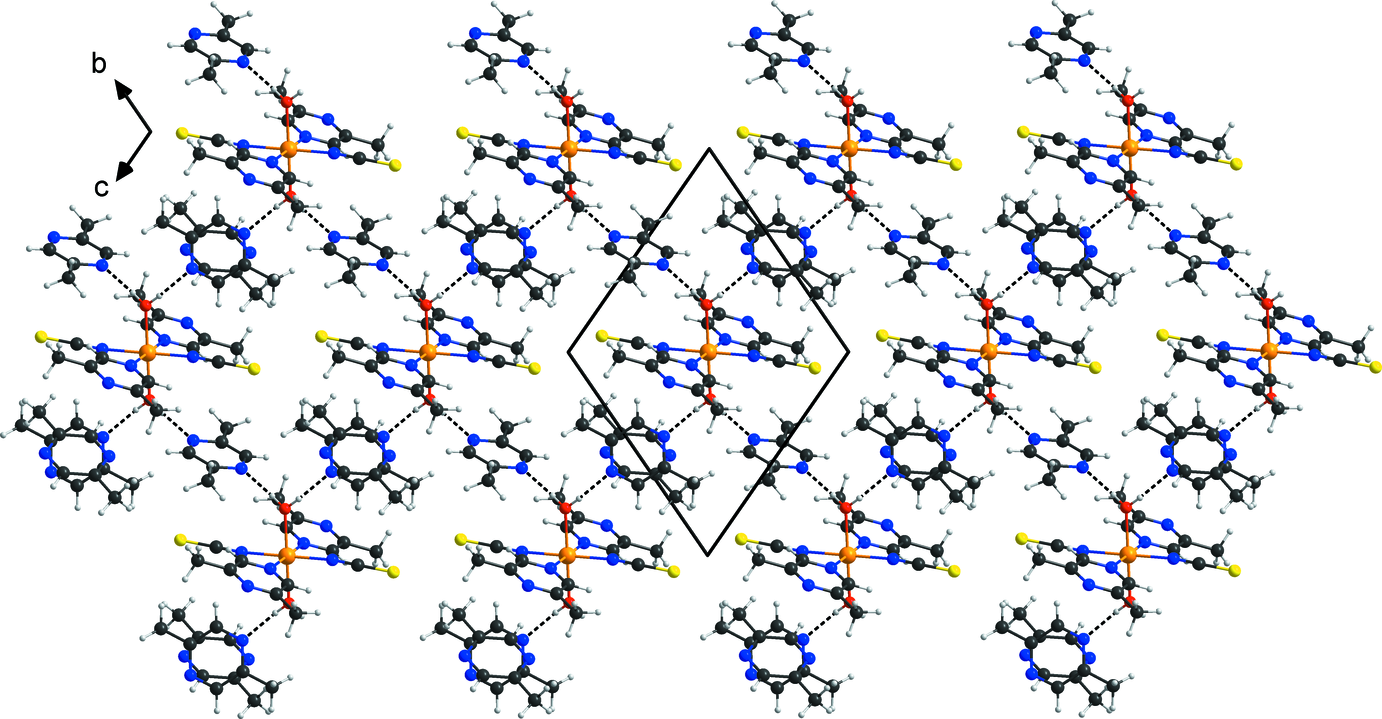

Supplement: Supplementary file 4 [file e-71-0m269-fig2.tif]
